# Facile and Rapid Formation of Giant Vesicles from Glass Beads

**DOI:** 10.3390/polym10010054

**Published:** 2018-01-09

**Authors:** Radu Tanasescu, Ute Mettal, Adai Colom, Aurélien Roux, Andreas Zumbuehl

**Affiliations:** 1 Department of Chemistry, University of Fribourg, Chemin du Musée 9, 1700 Fribourg, Switzerland; radu.tanasescu@unifr.ch (R.T.); ute.mettal@unifr.ch (U.M.); 2 Department of Biochemistry, University of Geneva, 30, Quai Ernest-Ansermet, 1211 Geneva, Switzerland; adai.colom@unige.ch (A.C.); aurelien.roux@unige.ch (A.R.); 3 National Centre of Competence in Research in Chemical Biology, 1211 Geneva, Switzerland

**Keywords:** formulation techniques, glass bead technique, giant vesicles, hybrid vesicles, phospholipids

## Abstract

Giant vesicles (GVs) are widely-used model systems for biological membranes. The formulation of these vesicles, however, can be problematic and artifacts, such as degraded molecules or left-over oil, may be present in the final liposomes. The rapid formulation of a high number of artifact-free vesicles of uniform size using standard laboratory equipment is, therefore, highly desirable. Here, the gentle hydration method of glass bead-supported thin lipid films has been enhanced by adding a vortexing step. This led to the formulation of a uniform population of giant vesicles. Batches of glass beads coated with different lipids can be combined to produce vesicles of hybrid lipid compositions. This method represents a stable approach to rapidly generate giant vesicles.

## 1. Introduction

Lipid vesicles or liposomes are 3D structures made from a bilayer of phospholipid molecules enclosing an aqueous compartment. Giant vesicles (GVs) typically have a diameter of >1 µm, which enables easy visualization and manipulation under an optical microscope. Today, many technologies exist to formulate lipid vesicles [1]. Most current methods have drawbacks, such as instability of molecules under the conditions used or residual solvent or oil left in the vesicles after preparation. What is needed is a simple and robust formulation technology that can be performed in any laboratory.

Giant vesicles can be formulated by adding an aqueous buffer to a dried thin film of lipids [2,3]. To accelerate this process, the film can be placed between two conductive plates and an applied alternating electrical field will promote the swelling of the lipid film into liposomes [4]. This method is now widely used and has been improved over the years for buffer exchange with flow chambers and for higher compatibility with respect to lipid and buffer compositions [5,6]. However, electroformation can lead to phospholipid degradation, thus limiting the type of lipids that can effectively be employed [7,8]. Furthermore, the method does not allow direct vesicle formulation under the high salt concentrations that would mimic a biological environment.

The agarose swelling method is based on spreading a lipid-containing solvent on a thin dried agarose film [9]. Upon buffer addition, GVs are generated at the swollen agarose/water interface. The speed of liposome formation is hereby highly increased compared to conventional hydration techniques and the method accommodates for a large range of lipid compositions and buffer conditions. Furthermore, it enables the efficient encapsulation of numerous biomolecules [9] including proteins [10]. The downside of this approach is that lipids are distributed over the entire gel thickness, which reduces the vesicle formation efficiency. Moreover, partial gel dissolution upon swelling leads to carry-over of the gel matrix into the vesicle sample [9]. An alternative is to use polyvinyl alcohol gels with optimized lipid distribution at the gel/buffer interface, which abolishes gel dissolution at room temperature [11]. However, at temperatures above 50 °C the gel starts to decompose, making the technique incompatible with the use of lipids having a high main phase transition temperature. A recent addition to the spectrum of GV preparation techniques is the formulation of GVs on cellulose paper [12].

Additionally, giant vesicles can be formed by creating double-emulsion droplets followed by oil removal [13], or by transferring lipid monolayer-stabilized droplets through an oil/water interface [14,15,16]. Encapsulating biomolecules into emulsion droplets, even at physiological conditions, is very effective and reproducible, and the technique can be implemented in microfluidic devices for high-throughput vesicle production [17,18]. However, drawbacks of the method include limited compatibility with a large repertoire of lipids (in part due to their poor solubility in the used solvent) and membrane contamination with traces of oil [10,19]. Yet, by applying the proper techniques, the oil contamination seems to be avoidable [20,21,22].

**Figure 1:**
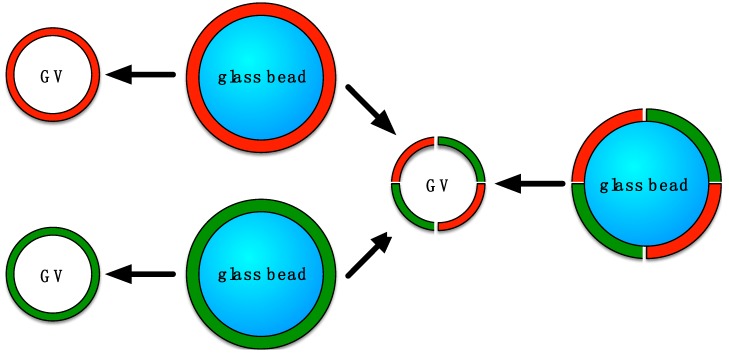
Schematic view of the formation of giant vesicles (GVs) by hydration of a glass-supported thin film of phospholipids while shaking. The glass beads are covered with thin multilayers of lipids, depicted in red and green. Two films containing two different types of phospholipids (either on the same bead or on different beads) can be combined into a hybrid vesicle.

Still, none of the GV formulation techniques is universal, and, e.g., there is a lack of uniform phospholipid bilayers for precise biophysical measurements [23]. Therefore, the first experiments with glass beads were initiated: small glass beads were vigorously shaken in a heated suspension of small vesicles. The liposomes broke up and solid supported bilayers formed on the glass surfaces [23]. Danelon and coworkers produced lipid-coated glass beads by directly adding the lipid-containing organic solvent onto the beads and evaporating the solvent [24]. The lipid-coated beads were then hydrated and giant unilamellar vesicles were formed. This method provides a much larger surface area of lipid film per volume compared to that with flat solid supports. In addition, lipid-coated beads can easily be aliquoted and the volume of the swelling solution can be as small as a few microliters. We reasoned that a concurrent shaking step would form more homogenous GVs because the generated vesicles should be sheared between the glass beads. Therefore, this method should be a robust, rapid, and convenient formulation technique for giant liposomes (see Figure 1).

## 2. Materials and Methods 

### 2.1. Materials

1,2-dipalmitoyl-*sn*-glycero-3-phosphocholine (DPPC) and 1,2-dioleoyl-*sn*-glycero-3-phosphocholine (DOPC) were purchased from Lipoid (Ludwigshafen, Germany) or Avanti Polar Lipids (Alabaster, AL, USA) and used without further purification. Laurdan [25,26] (6-dodecanoyl-*N*,*N*-dimethyl-2-naphthylamine) and Brand cavity slides with three cavities were purchased from Sigma-Aldrich (St. Louis, MO, USA). Glass beads, unwashed, 212–300 µm (50–70 U.S. sieve, average surface = 2.06 × 10^5^ μm^2^) were bought from Sigma-Aldrich (St. Louis, MO, USA). The glass beads were stirred in concentrated nitric acid for 2 h, washed with ultra-pure water followed by methanol and acetone, and dried under high vacuum (40 µbar) overnight.

### 2.2. Preparation of Glass Beads

The beads (2 g) were weighed in a round bottom glass flask and the appropriate volume (see paragraph “liposome formulation on glass beads”) of the lipid stock solution in chloroform (720 µg of lipid in 1 mL chloroform) was added on top plus 1 mL of chloroform to ensure optimal solvent coverage of the bead bed. For each experiment, a ratio of beads and lipids corresponding to a theoretical bead coverage with ten bilayers was used. For fluorescence imaging of the liposomes the membrane dye Laurdan (1 mol %) was included in the lipid mixture. The solvent was removed under reduced pressure using a rotary evaporator at mild rotation speed to ensure optimal coverage of the glass beads with the lipids. After complete solvent removal, the beads were dried under high vacuum overnight and stored at −20 °C under argon. The beads can be stored for at least one year (see supporting information Figure S1).

### 2.3. Liposome Formulation on Glass Beads

The different bead sizes qualitatively yielded the same results, therefore we focused on one bead size only, namely beads with an average size of 212–300 µm, corresponding to a surface of 1.4 × 10^5^–2.8 × 10^5^ µm^2^. For the formulation of GVs 200 mg of lipid-coated 212–300 µm-sized beads (corresponding to 72 µg of phospholipids) were weighed into a 2-mL Eppendorf vial. The appropriate swelling liquid, namely 500 µL of ultra-pure water (>18 MΩ·cm) or sucrose buffer (200 mM sucrose in phosphate buffered saline, pH 7.4, at an osmotic concentration of 400 mOsm/L) was added and the vial was shaken at 65 °C for 1 h at the indicated speed using a PHMT-PSC24 thermoshaker (Grant Bio, Cambridge, UK). Typically, only sucrose is used in GV formulation experiments. Here, phosphate-buffered saline was employed to create an environment of high ionic strength that inhibits vesicle formulation in many other techniques [7]. Prior to microscopy, 20 µL of the vesicle suspension prepared in sucrose buffer were added into the cavity of a Brand cavity slide and diluted five times with glucose buffer (200 mM glucose in phosphate buffered saline, pH 7.4, at an osmotic concentration of 400 mOsm/L). Vesicles formed in ultra-pure water were added onto the microscopy slide undiluted.

For the experiment where vesicles were formulated from different phospholipids on different glass beads, two different types of lipid-coated beads were prepared containing either DOPC or DPPC. Then 100 mg of each bead were mixed together in an Eppendorf tube with 500 µL hydrating liquid and the mixture was treated as stated above.

The first step of a typical vesicle preparation in a round bottom flask is the formation of a thin film. Slow evaporation of the solvent under rotation creates a visible lipid film on about half of the flask’s inner surface, i.e., 2.5 × 10^−3^ m^2^ for a 25-mL flask. Given a molecular size of 63 Å^2^ per DPPC molecule [27], the thin lipid film corresponds to roughly 1032 stacks of lipid bilayers. If this flask is half-filled with 212–300 µm-sized glass beads, the available area increases by two orders of magnitude. The thin lipid film now forms preferentially on the surface of the glass beads allowing for a thinner and more uniform lipid coating of the glass beads with theoretically ten stacks of bilayers. The dried lipid-coated glass beads can readily be aliquoted and stored in a freezer under inert atmosphere for at least one year without noticeable quality loss. It is also interesting to note that an amount of lipid-coated glass beads was used that corresponds to 72 µg of lipid. The standard thin film method would not allow working with such a low amount of lipid because hydration of the resulting thin film would require a too-small amount of buffer. 

During the formulation procedure, the vial containing the coated beads and the swelling liquid was heated above the main phase transition temperature of the corresponding lipid bilayers while simultaneously shaking the vial. We hypothesized that the shaking would influence the size of the GVs formed and that smaller diameter GVs would be formed when the speed of the shaking increases. Newly-formed vesicles would be mechanically reduced in diameter during the shaking step.

### 2.4. Preparation of Liposomes by Bead Gentle Hydration or by Electroformation

In order to compare the new method with other formulation techniques, the bead gentle hydration experiment was repeated following the standard protocol but omitting the shaking step.

For the electroformation experiments, a swelling chamber was constructed by placing a Teflon ring (17 mm inner diameter, 2 mm height) onto an ITO-coated microscopy slide (70–100 Ω/sq, Sigma-Aldrich, St. Louis, MO, USA). Then, 5 µL of a lipid solution (2 mg/mL) was spread into the Teflon ring. The organic solvent was removed under high vacuum for 1 h at 30 °C. The lipid film was hydrated with 500 µL of ultra-pure water or sucrose in PBS buffer and a second ITO-coated slide was positioned to close the chamber. Vesicles were grown by applying a sinusoidal wave (1 V and 10 Hz for 1 h at RT (DOPC) or 55 °C (DPPC or DPPC/DOPC-mixture)) using a potentiostat. For microscopy imaging 20 µL of the sucrose buffered vesicle suspension were diluted five times with glucose buffer. The vesicle suspensions formulated in ultra-pure water were used neat. 

### 2.5. Microscopy Experiments

For imaging, a Ti Eclipse confocal microscope (Nikon, Tokyo, Japan) was used with an excitation wavelength of 405 nm. The emitted light was split using a prism detector. The images were saved as Nikon Viewer software .nd2 files, exported as .tif files, and treated with Fiji/ImageJ software [28,29,30]. For visualization purposes, the image contrast was enhanced by 0.5%. In order to better discriminate between the gel and liquid crystalline membrane phases the Laurdan signal at 441 nm was artificially changed to red and the signal at 495 nm was artificially changed to green.

### 2.6. Generalized Polarization Experiment

The generalized polarization (*GP*) measurements were performed using the emission wavelengths at 441 nm (gel phase) and 495 nm (liquid crystalline phase). The resulting images were analyzed using the *GP*-plugin for Fiji/ImageJ [31]. The generalized polarization was calculated according to the formula:

$$GP=\frac{I_{441}-I_{495}}{I_{441}+I_{495}}$$

A high *GP* value would represent a liposomal gel phase membrane and a low *GP* represents a liposome membrane in a liquid crystalline phase [32].

## 3. Results and Discussion

### 3.1. Qualitative and Quantitative Comparison of Different GV Formulation Techniques

**Figure 2:**
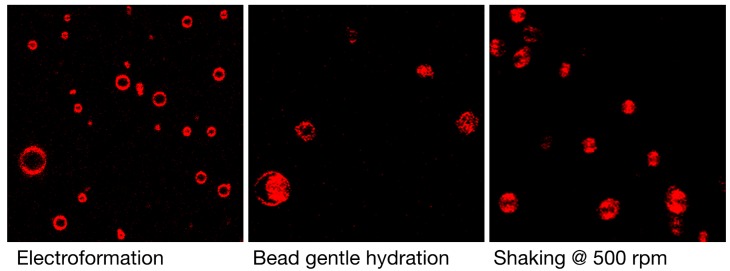
Typical qualitative perception of vesicle densities are represented in these low magnification images of a population of DPPC GVs formulated by different techniques in ultra-pure water. The glass bead shaking technique does produce a high density of vesicles. The sides of the quadratic figures measure 120 µm. The color was artificially changed to red. The individual vesicles are moving around leading to the shape effects seen in these pictures.

In addition to producing artifacts, many of the current GV preparation techniques suffer from the fact that a highly-polydispersed sample population is formed, slowing the process of finding suitable vesicle candidates for biophysical analysis. Figure 2 gives an overview of the typical density of giant vesicles formed by DPPC formulation in ultra-pure water. Shaking the glass beads during hydration leads to a similarly high density of vesicles under the microscope as found for the electroformation technique. 

**Figure 3:**
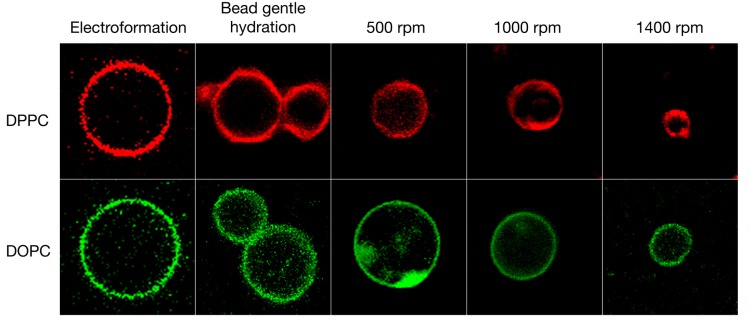
Giant vesicles formed from three different techniques: electroformation, gentle hydration of a thin film of lipids on glass beads, and the new method where the glass beads were vortex shaken during lipid film hydration at 65 °C for 1 h at different speeds. The vesicles were formulated from ultra-pure water. Each square is sized to 20 µm × 20 µm. The colors have been artificially adapted to red and green for bilayer gel and liquid crystalline phases, respectively.

In order to more quantitatively compare the different vesicle formulation techniques, DPPC and DOPC vesicles were prepared and their diameter and uniformity were assessed by measuring a series of liposomes (see Table 1). The two phospholipids will lead to GVs of different membrane fluidity, as seen by the emission of the co-formulated Laurdan probe: DPPC would be in a gel phase, whereas DOPC would be in a fluid liquid crystalline phase (see Figure 3). 

All the different techniques tested here yielded GVs in ultra-pure water (see Table 1). For DPPC vesicles, electroformation on ITO slides yielded GVs of 9.9 μm ± 4.9 μm in diameter and the gentle hydration of the glass beads without shaking yielded DPPC GVs of 8.8 μm ± 4.1 μm in diameter. Both methods yielded high differences in vesicle diameter of 4–5 μm. Combining the bead gentle hydration method with a shaking step significantly improved the uniformity of the vesicles and lowered the diameter differences to 1.3 μm or below. Furthermore, as anticipated, increasing the shaking speed by a factor of three during swelling significantly reduced the diameter of the GVs from 8.9 μm ± 1.3 μm at 500 rpm to 4.1 μm ± 1.1 μm at 1400 rpm possibly due to increased shearing of the GVs between the glass beads. Under the conditions used, the vesicles formed by electroformation were typically of larger diameter compared to the vesicles prepared by the other techniques. This trend was also visible for DOPC vesicles: electroformation yielded large GVs of 13.4 μm ± 5.6 μm diameter and gentle hydration lead to highly polydisperse GVs of 7.9 μm ± 7.2 μm diameter. Depending on the speed of shaking, the diameter of the GVs prepared by our new method ranged from 11.2 μm ± 2.2 μm at 500 rpm to 9.6 μm ± 1.2 μm at 1400 rpm each with a low polydispersity. The new technique, therefore, significantly simplifies access to GVs of desired sizes, independently of the phospholipid used.

### 3.2. GV Formulation in High Salt Conditions

**Figure 4:**
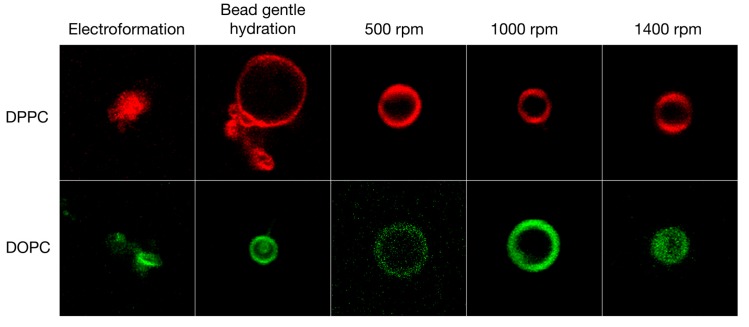
Giant vesicles formed in 400 mOsm/L PBS and a sucrose/glucose gradient. The shaking method was not affected by the high salt concentration. In contrast, electroformation did not yield vesicles. Each square is sized to 20 µm × 20 µm. The colors have been artificially adapted to red and green for bilayer gel and liquid crystalline phases, respectively.

Next, we formulated the same lipids again, but this time in a sucrose/glucose gradient at 400 mOsm/L PBS buffer concentration (see Figure 4). These high ionic strength conditions were deleterious for the electroformation method and no liposomes were formed anymore. This, of course, limits the usefulness of electroformation for experiments under biologically relevant conditions or requires reconstitution of the vesicle suspension with external buffer after formation in buffer-free conditions. The bead gentle hydration method was less affected by the high ionic strength and yielded GVs with medium diameters of 5.0 μm ± 2.6 μm for DPPC and 8.8 μm ± 4.9 μm for DOPC but high polydispersity (see Table 1). Compared to these methods, the shaking method was much less affected by the PBS present and yielded GVs of consistent diameters of 4.9 μm ± 0.9 μm for DPPC at 1400 rpm and 5.0 μm ± 1.0 μm for DOPC at 1400 rpm. Again, the diameter of the vesicles could be tuned by adapting the speed of the vortexing with speeds of 1400 rpm showing virtually no difference between GVs formulated in buffer-free and buffered solutions.

### 3.3. Formulation of GVs from Phospholipid Mixtures in Pure Water

**Figure 5:**
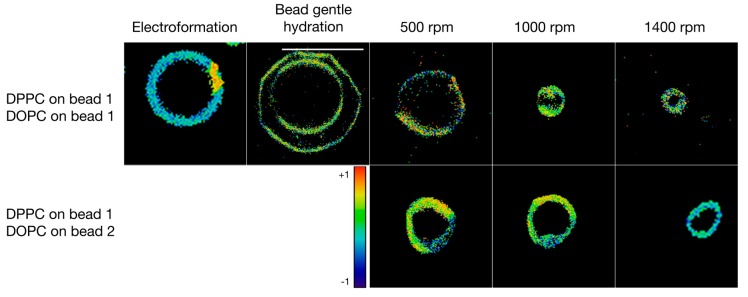
Giant vesicles formed in ultra-pure water. Each square but one is sized to 20 µm × 20 µm, the scale bar in one picture is 20 µm. The general polarization was calculated in order to quantify the extent of the gel vs. liquid crystalline phases (represented also in the heat map). The empty squares represent experiments that have been performed but did not yield mixed vesicles or that were not possible to perform using electroformation.

We then examined the formulation of GVs made from a 1:1 mixture of DPPC and DOPC. Figure 5 depicts the different GVs formed. First, the two lipids were weighed at equimolar concentration and subjected to the electroformation protocol. Additionally, the glass beads were coated with this mixture. The formulation led to hybrid GVs in ultra-pure water. The electroformation yielded GVs with large diameters and high polydispersity of 17.3 μm ± 9.1 μm (see Table 1). Bead gentle hydration yielded GVs of lower diameters but still high polydispersity of 9.2 μm ± 6.1 μm. Again, the vesicles formed by the shaking method were more uniform compared to the vesicles prepared with the other methods ranging from 10.4 μm ± 1.3 μm for 500 rpm shaking speed and 4.1 μm ± 1.2 μm for shaking at 1400 rpm (see Table 1). As seen in Figure 5, the GVs formed by bead gentle hydration showed a clear phase separation and the curvature difference between gel and liquid crystalline phases were clearly observed.

Unlike the other methods, where a lipid film of specific composition has to be prepared for each type of vesicles, our new approach opens the way for rapid formulation of various hybrid GVs. For this, we took equal weights of glass beads that were each coated with DPPC or DOPC and combined them in an Eppendorf tube. Bead gentle hydration of this mixture lead to the formulation of two separate populations of DPPC or DOPC GVs, each sized, respectively, at 5.3 μm ± 2.8 μm. In contrast to that, when the shaking step was added to the formulation procedure, hybrid liposomes formed at diameters between 8.3 μm ± 1.4 μm for 500 rpm and 4.6 μm ± 1.0 μm at 1400 rpm. This makes it possible to formulate GVs of various compositions starting from only a few batches of lipid-coated beads. Thus, glass beads coated with different thin lipid films can be combined to form hybrid GVs.

**Figure 6:**
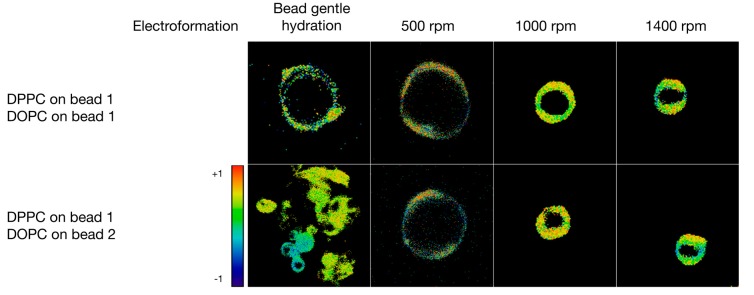
Calculated general polarization values for giant vesicles formulated in PBS buffer in a sucrose/glucose gradient. The empty squares represent experiments that were carried out, but did not yield vesicles or that were not possible to perform using electroformation. Each square is sized to 20 µm × 20 µm.

In another experiment, the hybrid vesicles were formulated at high ionic strength in a sucrose/glucose gradient (see Figure 6). Here, the bead gentle hydration did not yield consistent GVs. However, the shaking method yielded uniform vesicles and qualitatively, no difference between the GVs formulated from one bead versus two different beads was found. The mean diameters of the GVs ranged from 8.2 μm ± 1.3 μm at 500 rpm to 4.2 μm ± 0.7 μm at 1400 rpm shaking speed.

The possibility to combine different phospholipids into single hybrid vesicles just by changing the combination of a limited number of lipid-coated glass beads is, of course, quite powerful in terms of combinatorial potential but raises questions about how well the vesicles compare to vesicles formed from phospholipid combinations on a single glass bead. We, therefore, utilized the polarity-sensitive dye Laurdan in order to quantify membrane properties. Due to its sensitivity to the polarity of the immediate environment, Laurdan can be used to visualize changes in membrane fluidity [25,26]. Figure 5 and Figure 6 depict signals measured at the two Laurdan emission wavelengths of 441 nm (gel phase) and 495 nm (liquid crystalline phase). Using Laurdan instead of individual dedicated fluorescent membrane markers has the advantage that different types of phospholipids can be mixed together without the need for adapting the concentration of the marker molecules.

**Figure 7:**
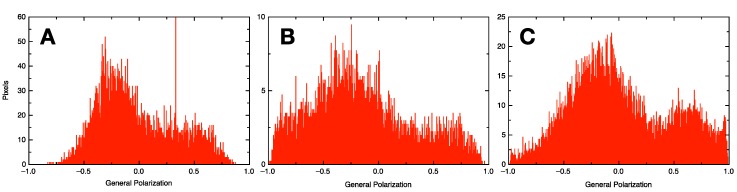
Comparison of the general polarization values measured from DPPC/DOPC GVs formulated via electroformation (**A**) or shaking method at 1400 rpm (**B**,**C**). For electroformation (**A**) a film of both DPPC and DOPC was formed in the electroformation chamber and formulated using pure water. For (**B**,**C**) two sets of glass beads were mixed, one coated with DPPC, the other with DOPC. The glass beads were hydrated in pure water (**B**) or in PBS buffer (**C**) with shaking at 1400 rpm and imaged in a sucrose/glucose gradient. Each histogram summarized an overview micrograph with at least 20 GVs. The three GV populations are qualitatively very similar.

Shifts in the emission wavelengths of Laurdan can be quantified in a generalized polarization analysis on the various GV combinations allowing a quantification of the extent of gel and liquid crystalline phases in each liposome ensemble [31,32]. The analysis was performed over all GV micrographs, averaging 20–300 individual GVs for each measurement. The measurement for mixed phospholipid GVs formulated by electroformation was taken as a reference (see Figure 7A). The GVs prepared by electroformation showed a higher content in liquid crystalline phase possibly due to the lower main phase transition temperature of DOPC compared to DPPC. 

The formulation using the shaking method yielded various results with preferences for either phospholipid (see Figures S2 and S3). Salt-free and high salt conditions could be combined with shaking at various speeds to formulate different GV populations. However, we found conditions where the resulting GV phospholipid composition was qualitatively very similar to the electroformation, although with a lower diameter (see Figure 7B,C). Interestingly, it was indeed possible to combine two sets of glass beads, each covered with a different phospholipid, and formulate a hybrid GV containing both lipids, provided the shaking speed during the hydration step was high enough. Globally, in water, there was a trend to form GVs with a preference for a liquid crystalline phase and, in high salt conditions, a gel phase was preferred. This trend was inversed by applying high shear forces.

## 4. Conclusions

We have presented a rapid formulation technique for monodisperse giant vesicles. Glass beads coated with dehydrated stacks of lipid bilayers can be conveniently prepared, stored at low temperature, and be easily distributed to other laboratories. Once needed, the glass beads are placed in an aqueous buffer solution and shaken at the appropriate speed and temperature. The initial coating of the glass beads with a thin lipid film requires a chemistry laboratory. After the solvent removal, no additional steps require any handling of organic solvents. The coated beads can be stored at low temperature for over one year. The quantities of lipids needed can be very low. Furthermore, beads covered with different lipids can be easily combined avoiding measuring very small amounts of hygroscopic phospholipids. After a one-hour hydration and vortexing step, giant vesicles are formed. This procedure offers facile access to hybrid GVs.

## Figures and Tables

**Table 1:** Quantitative comparison of the size and uniformity of GVs formed by different techniques in ultra-pure water or sucrose PBS buffer. If no value is recorded, then the corresponding experiments have been carried out, but no liposomes were formed.

Lipid	Solvent	Formulation	#GVs Counted	Size ± STD (μm)
DPPC	water	Electroformation	24	9.9 ± 4.9
		Bead gentle hydration	11	8.8 ± 4.1
		Shaking @ 500 rpm	14	8.9 ± 1.3
		Shaking @ 1000 rpm	26	7.8 ± 1.3
		Shaking @ 1400 rpm	21	4.1 ± 1.1
DPPC	PBS sucrose	Electroformation	- ^1^	- ^1^
		Bead gentle hydration	20	5.0 ± 2.6
		Shaking @ 500 rpm	15	7.5 ± 1.2
		Shaking @ 1000 rpm	20	4.9 ± 1.1
		Shaking @ 1400 rpm	23	4.9 ± 0.9
DOPC	water	Electroformation	19	13.4 ± 5.6
		Bead gentle hydration	26	7.9 ± 7.2
		Shaking @ 500 rpm	17	11.2 ± 2.2
		Shaking @ 1000 rpm	23	9.6 ± 1.6
		Shaking @ 1400 rpm	25	5.9 ± 1.2
DOPC	PBS sucrose	Electroformation	- ^1^	- ^1^
		Bead gentle hydration	13	8.8 ± 4.9
		Shaking @ 500 rpm	21	7.8 ± 1.5
		Shaking @ 1000 rpm	19	6.6 ± 1.2
		Shaking @ 1400 rpm	16	5.0 ± 1.0
DPPC on bead 1 DOPC on bead 1	water	Electroformation	31	17.3 ± 9.1
		Bead gentle hydration	23	9.2 ± 6.1
		Shaking @ 500 rpm	24	10.4 ± 1.3
		Shaking @ 1000 rpm	14	5.9 ± 1.4
		Shaking @ 1400 rpm	19	4.1 ± 1.2
DPPC on bead 1 DOPC on bead 1	PBS sucrose	Electroformation	- ^1^	- ^1^
		Bead gentle hydration	11	6.9 ± 2.3
		Shaking @ 500 rpm	19	10.8 ± 1.3
		Shaking @ 1000 rpm	21	6.1 ± 1.1
		Shaking @ 1400 rpm	19	4.6 ± 0.6
DPPC on bead 1 DOPC on bead 2	water	Electroformation	- ^2^	- ^2^
		Bead gentle hydration	29	5.3 ± 2.8
		Shaking @ 500 rpm	16	8.3 ± 1.4
		Shaking @ 1000 rpm	17	7.8 ± 1.3
		Shaking @ 1400 rpm	21	4.6 ± 1.0
DPPC on bead 1 DOPC on bead 2	PBS sucrose	Electroformation	- ^2^	- ^2^
		Bead gentle hydration	21	4.7 ± 2.7
		Shaking @ 500 rpm	18	8.2 ± 1.3
		Shaking @ 1000 rpm	16	5.6 ± 1.1
		Shaking @ 1400 rpm	24	4.2 ± 0.7

^1^ No GVs formed in the experiments; ^2^ This experiment cannot be performed using electroformation.

## Supplementary Materials

The following are available online at www.mdpi.com/2073-4360/10/1/54/s1, Figure S1: GV formulated from 1 year old coated beads. Figures S2 and S3: complete set of general polarization histograms measured in water and buffer.
