# Supplementary material for: Facile and Rapid Formation of Giant Vesicles from Glass Beads
Source: Polymers (Basel). 2018 Jan 9;10(1):54. doi: 10.3390/polym10010054 (PMC6414939; doi:10.3390/polym10010054)
Supplement: Supplementary file 1 [file polymers-10-00054-s001.pdf]

Supporting Information for

Article

Facile and Rapid Formation of Giant Vesicles from Glass Beads

Radu Tanasescu [1], Ute Mettal [1], Adai Colom [2,3], Aurélien Roux [2,3] and Andreas Zumbuehl [1,3,*]

[1] Department of Chemistry, University of Fribourg, Chemin du Musée 9, 1700 Fribourg, Switzerland; radu.tanasescu@unifr.ch (R.T.); ute.mettal@unifr.ch (U.M.)
[2] Department of Biochemistry, University of Geneva, 30, Quai Ernest-Ansermet, 1211 Geneva, Switzerland; adai.colom@unige.ch (A.C.); aurelien.roux@unige.ch (A.R.)
[3] National Centre of Competence in Research in Chemical Biology, 1211 Geneva, Switzerland
* Correspondence: andreas.zumbuehl@unifr.ch; Tel.: +41-26-300-8794

Glass beads used after 1 year in the freezer

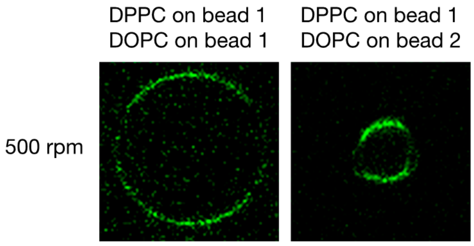

Figure S1. Representative confocal micrographs of DPPC/DOPC vesicles hydrated at 65 °C and 500 rpm from glass beads that stayed 1 year in the freezer at −20 °C. Each square is sized to 20 µm × 20 µm. The micrographs were recorded using 1 wavelength only, therefore no general polarization image is provided. The vesicles were artificially colored green for increased visibility.

Complete set of general polarization histograms

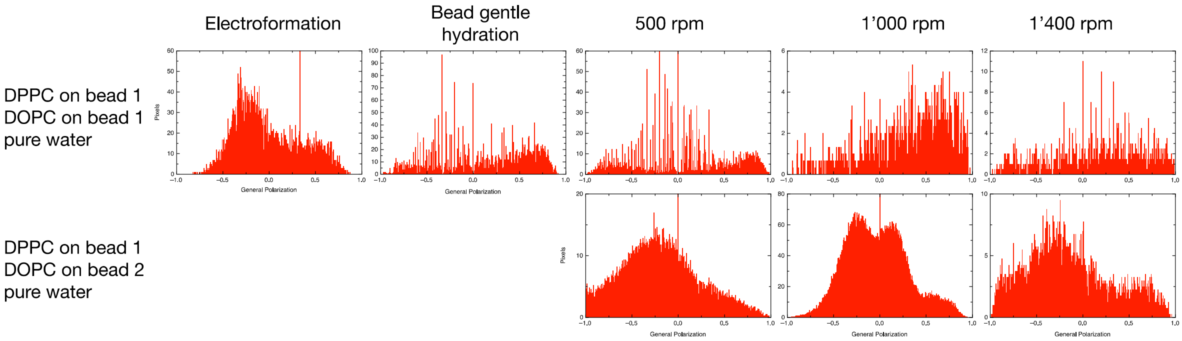

Figure S2. Complete set of general polarization histograms of vesicles formulated in pure water calculated from overview micrographs of the GV. Each histogram summarizes at least 20 GVs.

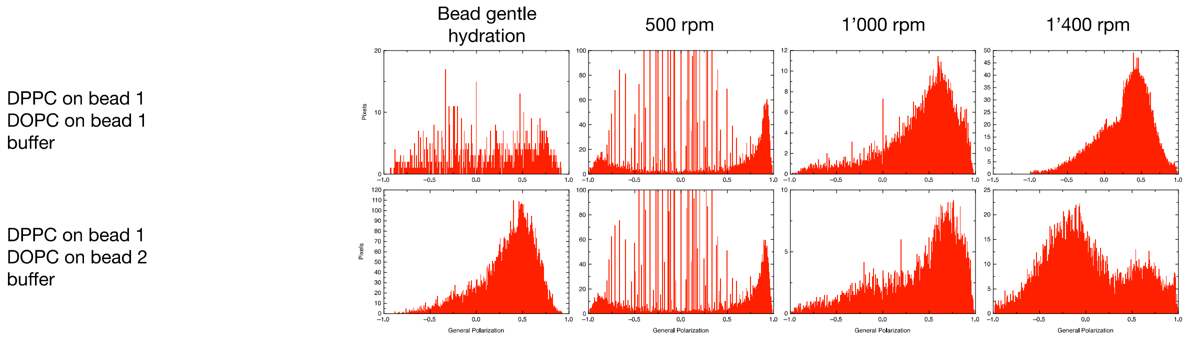

Figure S1. Complete set of general polarization histograms of vesicles formulated in PBS buffer in a sucrose/glucose gradient. The data were calculated from overview micrographs of the GV. Each histogram summarizes at least 20 GVs.
